# Supplementary material for: Effects of light curing on silver diamine fluoride-treated carious lesions: A systematic review
Source: PLoS One. 2024 Aug 12;19(8):e0306367. doi: 10.1371/journal.pone.0306367 (PMC11318914; doi:10.1371/journal.pone.0306367)
Supplement: S1 Table — (DOCX) [file pone.0306367.s002.docx]

**Table S 1. Search Strategy**

**PubMed Library**

| **Domain** | **No** | **Search Terms (All fields)** | **Total** |
| --- | --- | --- | --- |
| **Population (P)** | 1  2  3  4  5 | Carious lesion  Dental caries  Dentin or dentine  Enamel  1 or 2 or 3 or 4 | 65750  65418  42827  39482  123,105 |
| **Intervention (I)** | 4  5  6  7  8 | Dental light curing  Dental curing light  LED curing  Dental laser  Dental polymerization  4 or 5 or 6 or 7 or 8 | 5965  3596  2605  13661  64010  78058 |
| **Comparator (C)** | 8  9  10  11 | Silver diamine fluoride  Silver fluoride  SDF  Silver fluoride diamine  8 or 9 or 10 or 11 | 555  164  8266  62  8524 |
| **PICO** |  | **P and I and C** | **80** |
| **Scopus** | 1  2  3 | Silver diamine fluoride or SDF or Silver fluoride or silver fluoride diamine  Carious lesion or dental caries or enamel or dentine or dentin  Dental light curing or dental curing light or LED curing or Dental laser or Dental polymerization  **1 or 2 or 3** | 3988  66476  10054  **20** |
| **EBSCO** | 4  5  6 | Silver diamine fluoride or SDF or Silver fluoride or silver fluoride diamine  Carious lesion or dental caries or enamel or dentine or dentin  Dental light curing or dental curing light or LED curing or Dental laser or Dental polymerization  **4 or 5 or 6** | 602  60037  11352  **12** |
| **Google scholar** |  | Silver diamine fluoride must include light curing. | **63** |

silver diamine fluoride: "silver diamine fluoride"[All Fields], "silver fluoride [All Fields]

carious lesions: "dental caries"[MeSH Terms]

dentin: "dentin"[MeSH Terms]

dental curing lights: "curing lights, dental"[MeSH Terms]

light: "light"[MeSH Terms]

laser: "laser's"[All Fields] OR "lasers"[MeSH Terms]
