# Supplementary material for: Effects of light curing on silver diamine fluoride-treated carious lesions: A systematic review
Source: PLoS One. 2024 Aug 12;19(8):e0306367. doi: 10.1371/journal.pone.0306367 (PMC11318914; doi:10.1371/journal.pone.0306367)
Supplement: Attachment — Submitted filename: Rebuttal letter.docx [file pone.0306367.s003.docx]

Dear Dr. Deepavali A Dhond,

We wish to express our appreciation to you and the reviewers for your valuable time and effort in reviewing our manuscript entitled "Effects of Light Curing on Silver Diamine Fluoride-Treated Carious Lesions: A Systematic Review" (Ref: PONE-D-23-25480).

The recommendations and advice provided have significantly contributed to the improvement of our manuscript's quality. In accordance with your recommendations, we have applied the QUIN tool, a dedicated risk of bias assessment tool designed specifically for in vitro studies. This assessment is detailed in Table 1 on Page 5, while the corresponding quality assessment table is provided on Page 10 (Table 4).This tool has been utilized to assess the risk of bias in studies, particularly those conducted within the field of dentistry.

All authors have read and approved the revised manuscript. We hope that our resubmission is now suitable for inclusion in PLOS ONE journal and we look forward to hearing from you.

Sincerely Yours,

Palinee Detsomboonrat

**E-mail**	[Palinee.d@chula.ac.th](mailto:Palinee.d@chula.ac.th)
