# Supplementary material for: Effects of light curing on silver diamine fluoride-treated carious lesions: A systematic review
Source: PLoS One. 2024 Aug 12;19(8):e0306367. doi: 10.1371/journal.pone.0306367 (PMC11318914; doi:10.1371/journal.pone.0306367)
Supplement: Attachment — Submitted filename: Response to Reviewers.docx [file pone.0306367.s004.docx]

**“Response to Reviewers”**

Journal requirements:

1.When submitting your revision, we need you to address these additional requirements.

Please ensure that your manuscript meets PLOS ONE's style requirements, including those for file naming. The PLOS ONE style templates can be found at

https://journals.plos.org/plosone/s/file?id=wjVg/PLOSOne_formatting_sample_main_body.pdf and

https://journals.plos.org/plosone/s/file?id=ba62/PLOSOne_formatting_sample_title_authors_affiliations.pdf.

Response: We edited the manuscript meets PLOS ONE's style requirements

2. Note from Emily Chenette, Editor in Chief of PLOS ONE, and Iain Hrynaszkiewicz, Director of Open Research Solutions at PLOS: Did you know that depositing data in a repository is associated with up to a 25% citation advantage (https://doi.org/10.1371/journal.pone.0230416)? If you’ve not already done so, consider depositing your raw data in a repository to ensure your work is read, appreciated and cited by the largest possible audience. You’ll also earn an Accessible Data icon on your published paper if you deposit your data in any participating repository (https://plos.org/open-science/open-data/#accessible-data).

3. We note that the grant information you provided in the ‘Funding Information’ and ‘Financial Disclosure’ sections do not match.

When you resubmit, please ensure that you provide the correct grant numbers for the awards you received for your study in the ‘Funding Information’ section.

Response: We edited ‘Funding Information’ and ‘Financial Disclosure’ sections

4. Thank you for stating the following financial disclosure:

[This research project is supported by the Second Century Fund (C2F), Chulalongkorn University.].

Please state what role the funders took in the study. If the funders had no role, please state: ""The funders had no role in study design, data collection and analysis, decision to publish, or preparation of the manuscript.""

If this statement is not correct you must amend it as needed.

Please include this amended Role of Funder statement in your cover letter; we will change the online submission form on your behalf.

Response: We added state: "The funders had no role in study design, data collection and analysis, decision to publish, or preparation of the manuscript." in cover letter.

5. Thank you for stating the following in the Acknowledgments Section of your manuscript:

[This research project is supported by the Second Century Fund (C2F), Chulalongkorn University.].

We note that you have provided funding information that is not currently declared in your Funding Statement. However, funding information should not appear in the Acknowledgments section or other areas of your manuscript. We will only publish funding information present in the Funding Statement section of the online submission form.

Please remove any funding-related text from the manuscript and let us know how you would like to update your Funding Statement. Currently, your Funding Statement reads as follows:

[This research project is supported by the Second Century Fund (C2F), Chulalongkorn University.].

Please include your amended statements within your cover letter; we will change the online submission form on your behalf.

Response: We removed any funding-related text from the manuscript

[Note: HTML markup is below. Please do not edit.]

Reviewers' comments:

Reviewer's Responses to Questions

Comments to the Author

1. Is the manuscript technically sound, and do the data support the conclusions?

The manuscript must describe a technically sound piece of scientific research with data that supports the conclusions. Experiments must have been conducted rigorously, with appropriate controls, replication, and sample sizes. The conclusions must be drawn appropriately based on the data presented.

Reviewer #1: Partly

2. Has the statistical analysis been performed appropriately and rigorously?

Reviewer #1: N/A

3. Have the authors made all data underlying the findings in their manuscript fully available?

The PLOS Data policy requires authors to make all data underlying the findings described in their manuscript fully available without restriction, with rare exception (please refer to the Data Availability Statement in the manuscript PDF file). The data should be provided as part of the manuscript or its supporting information, or deposited to a public repository. For example, in addition to summary statistics, the data points behind means, medians and variance measures should be available. If there are restrictions on publicly sharing data—e.g. participant privacy or use of data from a third party—those must be specified.

Reviewer #1: Yes

4. Is the manuscript presented in an intelligible fashion and written in standard English?

PLOS ONE does not copyedit accepted manuscripts, so the language in submitted articles must be clear, correct, and unambiguous. Any typographical or grammatical errors should be corrected at revision, so please note any specific errors here.

Reviewer #1: No

5. Review Comments to the Author

Please use the space provided to explain your answers to the questions above. You may also include additional comments for the author, including concerns about dual publication, research ethics, or publication ethics. (Please upload your review as an attachment if it exceeds 20,000 characters)

Reviewer #1: The abstract lacks specific details regarding the date of data collection and the range of languages examined—whether it includes articles solely in English or those in other languages as well.

Response: We added the date of data collection and the range of languages examined in the abstract (Page 1 Line 8-9).

Additionally, there is an inconsistency in the presentation of authors' initials within the methodology section.

Response: We checked the presentation of authors' initials within the methodology section consistently.

The term "in vitro" should be italicized throughout the document for proper emphasis.

Response: We changed "in vitro" to be “*in vitro*” throughout the document.

Drawing a conclusion about the potentiation of antimicrobial effects by light based solely on a single referenced article is insufficient and lacks robust support.

Response: We understand and acknowledge your point that relying solely on one reference may not provide robust support for our conclusion. Upon reflection, we agree that a more comprehensive analysis incorporating multiple studies would enhance the credibility and robustness of our findings. To address this concern, we have revised the conclusion section to regard the potential potentiation of antimicrobial effects by light in conjunction with SDF treatment, based solely on a single referenced article. It also underscores the importance of further research to validate these findings in clinical settings (Page 16 Line 223-227).

Furthermore, in the table, distinct sections should be allocated for studies assessing microhardness, antimicrobial effects, and other effects of Silver Diamine Fluoride (SDF).

Response: In Table 3, distinct sections have been allocated for studies assessing the micro hardness, antimicrobial effects, and other effects of Silver Diamine Fluoride (SDF) in outcome column. However, the author has reordered it so that studies with similar outcomes are grouped.

The entire text requires thorough editing to address these language and grammatical inaccuracies.

Response: The entire text was edited to address these language and grammatical inaccuracies by the Papercheck. We also attached the certificate of editing.

6. PLOS authors have the option to publish the peer review history of their article (what does this mean?). If published, this will include your full peer review and any attached files.

If you choose “no”, your identity will remain anonymous but your review may still be made public.

Do you want your identity to be public for this peer review? For information about this choice, including consent withdrawal, please see our Privacy Policy.

Reviewer #1: No

[NOTE: If reviewer comments were submitted as an attachment file, they will be attached to this email and accessible via the submission site. Please log into your account, locate the manuscript record, and check for the action link "View Attachments". If this link does not appear, there are no attachment files.]

While revising your submission, please upload your figure files to the Preflight Analysis and Conversion Engine (PACE) digital diagnostic tool, https://pacev2.apexcovantage.com/. PACE helps ensure that figures meet PLOS requirements. To use PACE, you must first register as a user. Registration is free. Then, login and navigate to the UPLOAD tab, where you will find detailed instructions on how to use the tool. If you encounter any issues or have any questions when using PACE, please email PLOS at figures@plos.org. Please note that Supporting Information files do not need this step.

Response: We uploaded our figure file to PACE digital diagnostic tool and edited.
