# Supplementary material for: Effects of light curing on silver diamine fluoride-treated carious lesions: A systematic review
Source: PLoS One. 2024 Aug 12;19(8):e0306367. doi: 10.1371/journal.pone.0306367 (PMC11318914; doi:10.1371/journal.pone.0306367)
Supplement: Attachment — Submitted filename: Response2 to Reviewers.docx [file pone.0306367.s005.docx]

**Response to Reviewers (9/5/2024)**

PONE-D-23-29346R1

Effects of light curing on silver diamine fluoride-treated carious lesions: A systematic review

Dear Reviewer,

We have addressed your concerns and made revisions accordingly. Below is our detailed response to each of the points raised:

1. Purpose and Research Question

Reviewer Comment: The research question needs to be clearly stated.

Response: Thank you for pointing this out. We have revised the manuscript to clearly articulate our research question. The research question now reads: "Does the combination of silver diamine fluoride (SDF) and light curing enhance the effectiveness of SDF in treating carious lesions in primary teeth compared to SDF alone?" This has been updated in the Introduction section in Line 72-75

2. Lack of Consistent Argument and Mechanism of Action

Reviewer Comment: The study lacks a consistent argument for the combination of SDF and light curing. The mechanism by which light curing could improve the effectiveness of SDF is not well-explained, and the conclusions drawn are not robust.

Response: We expand the Discussion section to include a more comprehensive review of the potential mechanisms and add references. Specifically, we discuss the hypothesis that light exposure could alter the shape and activity of silver particles, as suggested by Karnowakul et al. (2022). We also elaborate on the possible interactions between light and SDF that might contribute to increased mineralization and antimicrobial activity. Nevertheless, due to the limited number of studies with a lack of clinical studies to support the effectiveness, we acknowledge that the mechanism behind is not yet fully understood and a robust conclusion cannot be drawn.

3. Justification for Systematic Review

Reviewer Comment: Authors claim that there is no systematic review (SR) related to this subject, but this alone is not a sufficient justification for the study.

Response: We have revised the Introduction to better justify the significance of our systematic review in Line 79-85. We emphasize the clinical relevance of exploring adjunctive treatments to enhance the efficacy of SDF, the growing interest in non-invasive dental treatments, and the potential implications for dental care. We also highlight the gap in the literature regarding the combination of SDF and light curing, which our study aims to address.

4. In Vitro Studies and Evidence Robustness

Reviewer Comment: Only in vitro studies were found according to the criteria, with the majority (4 out of 5) tested on primary teeth. The study lacks robust evidence for the combination of SDF and light curing.

Response: Agree, we add your valuable comment to the manuscript in line 233-235. “due to the limited number of five in-vitro studies (4 out of 5 tested on primary teeth) and a lack of clinical studies, the mechanism of using Light curing to improve the clinical outcomes is not yet fully understood. Therefore, a robust conclusion cannot be drawn.”

We have clarified the limitations of our systematic review, particularly the reliance on in vitro studies, in the Limitations section in Line 249-250. We discuss the implications of these limitations for the generalizability of our findings to clinical practice and emphasize the need for future clinical trials to establish robust evidence in Line 250-254. Additionally, we have provided a more detailed summary of the in vitro studies included, highlighting their methodologies, findings, and relevance to the research question in line 256-259.

5. References and Specific Studies

Reviewer Comment: Specific studies (references 15, 19, and 20) are cited, but their relevance and mechanisms are not well explained.

Response: We have revisited the referenced studies and provided a more detailed discussion of their findings and relevance to our research. In the revised manuscript, we explicitly discuss how each study contributes to our understanding of the potential benefits and mechanisms of combining SDF with light curing in Line 201-203, 215-228. We also critically analyze the limitations of these studies and how they inform our systematic review in Line 228-235.

We hope that these revisions satisfactorily address your concerns. We believe that the changes made have strengthened our manuscript, and we appreciate the opportunity to improve our work based on your insightful feedback.

Thank you for your time and consideration.

Sincerely,
